# A contemporary training concept in critical care cardiology

**DOI:** 10.3389/fcvm.2024.1351633

**Published:** 2024-03-14

**Authors:** Leonhard Binzenhöfer, Nils Gade, Daniel Roden, Inas Saleh, Hugo Lanz, Laura Villegas Sierra, Paula Seifert, Clemens Scherer, Benedikt Schrage, Franz Haertel, Peter M. Spieth, Norman Mangner, Christoph Adler, Daniel Hoyer, Tobias Graf, Hannah Billig, Mostafa Salem, Rafael Henrique Rangel, Walter S. Speidl, Christian Hagl, Jörg Hausleiter, Steffen Massberg, Michael Preusch, Benjamin Meder, David M. Leistner, Peter Luedike, Tienush Rassaf, Sebastian Zimmer, Dirk Westermann, Uwe Zeymer, Andreas Schäfer, Holger Thiele, Enzo Lüsebrink

**Affiliations:** ^1^ Department of Medicine I, LMU University Hospital, LMU Munich and DZHK (German Center for Cardiovascular Research), Partner Site Munich Heart Alliance, Munich, Germany; ^2^ Department of Cardiology, University Heart and Vascular Center Hamburg and DZHK (German Center for Cardiovascular Research), Partner Site Hamburg/Kiel/Lübeck, Hamburg, Germany; ^3^ Klinik für Innere Medizin I, Universitätsklinikum Jena, Jena, Germany; ^4^ Klinik und Poliklinik für Anästhesiologie und Intensivtherapie, Universitätsklinikum Carl Gustav Carus an der Technischen Universität Dresden, Dresden, Germany; ^5^ Klinik für Innere Medizin und Kardiologie, Herzzentrum Dresden, Technische Universität Dresden, Dresden, Germany; ^6^ Klinik III für Innere Medizin, Herzzentrum der Universität zu Köln, Köln, Germany; ^7^ Universitätsklinik und Poliklinik für Innere Medizin III Kardiologie, Angiologie und Internistische Intensivmedizin, Universitätsklinikum Halle (Saale), Halle (Saale), Germany; ^8^ Medizinische Klinik II (Kardiologie, Angiologie und Intensivmedizin), Universitätsklinikum Schleswig-Holstein and DZHK (German Center for Cardiovascular Research), Partner Site Hamburg, Kiel, Lübeck, Germany; ^9^ Department of Internal Medicine II, Heart Center Bonn, University Hospital Bonn, Bonn, Germany; ^10^ Klinik für Innere Medizin III, Universitätsklinikum Schleswig-Holstein, Kiel and DZHK (German Center for Cardiovascular Research), Partner Site Hamburg, Kiel, Lübeck, Germany; ^11^ Division of Cardiology, Department of Internal Medicine II, Medical University of Vienna, Vienna, Austria; ^12^ Department of Cardiac Surgery, LMU University Hospital, LMU Munich and DZHK (German Center for Cardiovascular Research), Partner Site Munich Heart Alliance, Munich, Germany; ^13^ Department of Cardiology, Angiology, and Pneumology, University Hospital Heidelberg, Heidelberg, Germany; ^14^ Department of Cardiology and Angiology, University Hospital Frankfurt, Frankfurt, Germany; ^15^ Department of Cardiology and Vascular Medicine, West German Heart and Vascular Center, University Hospital Essen, Essen, Germany; ^16^ Department of Cardiology and Angiology, Medical Center, University of Freiburg, Freiburg, Germany; ^17^ Klinik für Kardiologie, Pneumologie, Angiologie und internistische Intensivmedizin, Klinikum der Stadt Ludwigshafen and Institut für Herzinfarktforschung, Ludwigshafen am Rhein, Germany; ^18^ Department of Cardiology and Angiology, Hannover Medical School, Hannover, Germany; ^19^ Department of Internal Medicine/Cardiology and Leipzig Heart Science, Heart Center Leipzig at University of Leipzig, Leipzig, Germany

**Keywords:** critical care cardiology, training concept, core curriculum, intensive care unit, cardiovascular fellows, fellows in training

## Abstract

Critical care cardiology (CCC) in the modern era is shaped by a multitude of innovative treatment options and an increasingly complex, ageing patient population. Generating high-quality evidence for novel interventions and devices in an intensive care setting is exceptionally challenging. As a result, formulating the best possible therapeutic approach continues to rely predominantly on expert opinion and local standard operating procedures. Fostering the full potential of CCC and the maturation of the next generation of decision-makers in this field calls for an updated training concept, that encompasses the extensive knowledge and skills required to care for critically ill cardiac patients while remaining adaptable to the trainee’s individual career planning and existing educational programs. In the present manuscript, we suggest a standardized training phase in preparation of the first ICU rotation, propose a modular CCC core curriculum, and outline how training components could be conceptualized within three sub-specialization tracks for aspiring cardiac intensivists.

## Introduction

**Figure 1:**
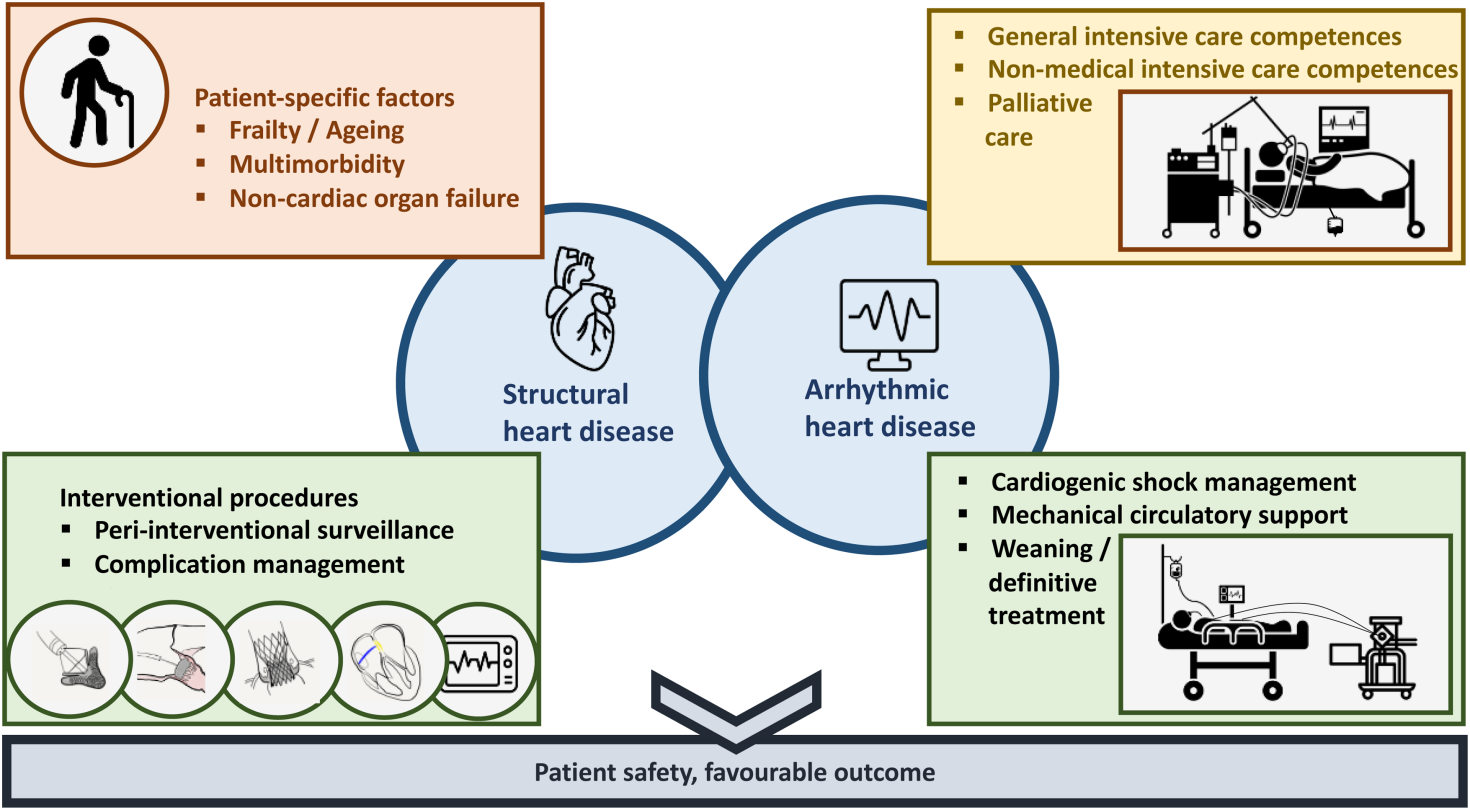
Challenges of contemporary critical care cardiology.

Critical care cardiology (CCC) in the modern era is shaped by a multitude of innovative treatment options and an increasingly complex, ageing patient population (Figure 1) (1–3). Generating high-quality evidence for novel interventions and devices in an intensive care setting is exceptionally challenging. As a result, formulating the best possible therapeutic approach continues to rely predominantly on expert opinion and local standard operating procedures. Fostering the full potential of CCC and the maturation of the next generation of decision-makers in this field calls for an updated training concept, that encompasses the extensive knowledge and skills required to care for critically ill cardiac patients while remaining adaptable to the trainee's individual career planning and existing educational programs (4–7). In the present manuscript, we suggest a standardized training phase in preparation of the first intensive care unit (ICU) rotation, propose a modular CCC core curriculum, and outline how training components could be conceptualized within three sub-specialization tracks for aspiring cardiac intensivists.

## Preparation for critical care training

**Figure 2:**
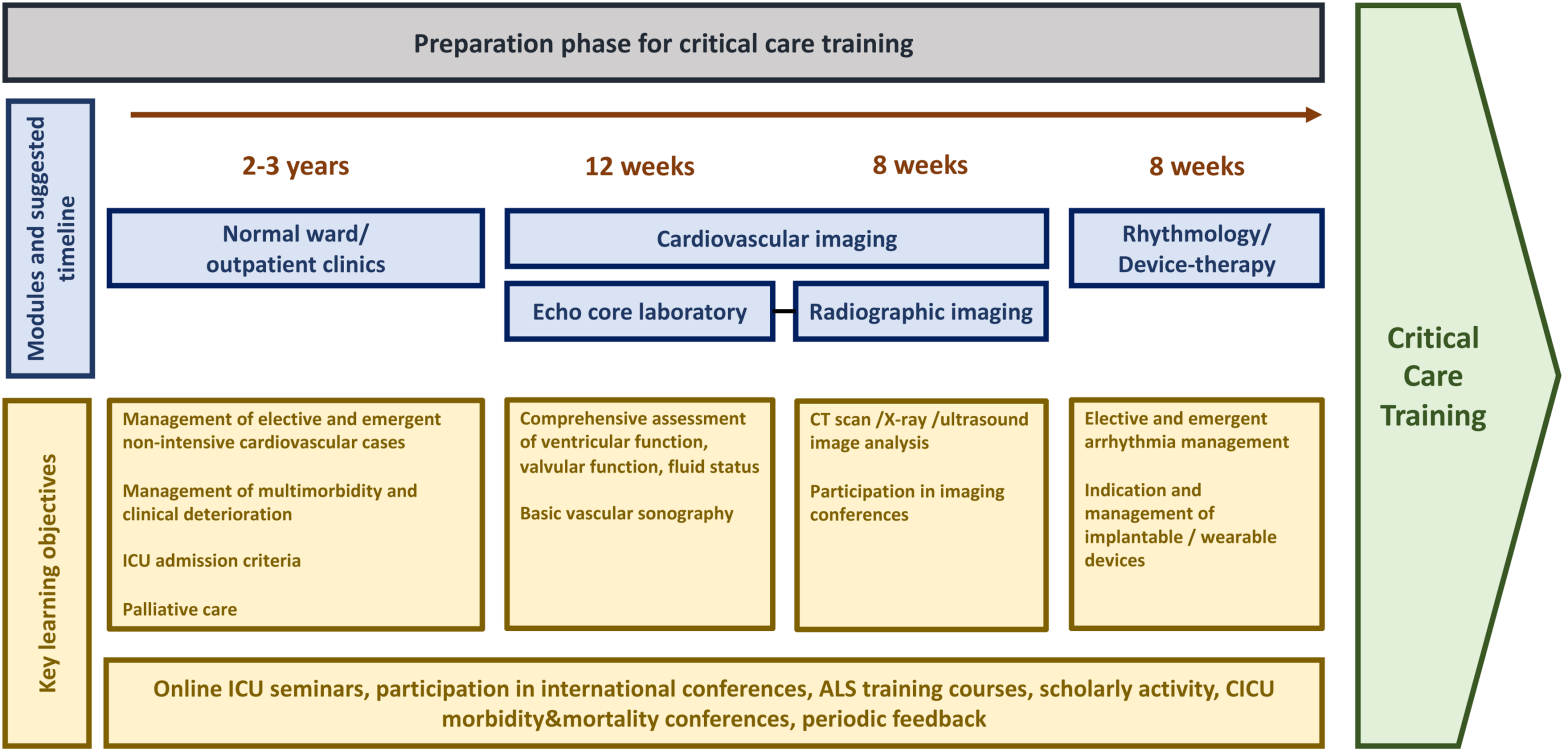
Preparation for critical care training. ALS, advanced life support; CICU, cardiac intensive care unit; CT, computed tomography; ICU, intensive care unit.

Cardiovascular fellows must be prepared for their first intensive care training phase in the best possible way during the preceding internal medicine/cardiovascular training program. Our exemplary proposal for a standardized pre-intensive care unit (pre-ICU) training comprises basic cardiovascular training elements with an intermediate period of focused practical skill acquisition and off-patient theoretical ICU preparation, leading up to the first supervised phases of intensive care training (Figure 2). Although many institutions may already have a pre-ICU training concept in place, we advocate for including standard prerequisites for a first ICU rotation into a comprehensive CCC training model.

Trainees entering the internal medicine department usually spend 2–3 years primarily working in elective and emergency non-intensive care scenarios on a normal ward, outpatient clinic, or emergency department. Multimorbidity and clinical deterioration, however, may be encountered early on and offer learning opportunities in organ-specific disease management, patient selection for various procedures, ICU admission criteria, and palliative care. Thereafter, training programs should provide cardiovascular fellows scheduled for/interested in (cardiac) intensive care with the opportunity to train in a high-throughput echocardiography laboratory for at least twelve weeks. Valvular function analysis, comprehensive right and left ventricular assessment, and evaluation of fluid status and effusions should be trained extensively and under supervision of a senior clinician to allow for focussed investigation even in suboptimal conditions and emergency settings in the cardiac ICU. Crude assessment of peripheral vessels to exclude arterial occlusion, active access site bleeding or arteriovenous fistulas, as well as thromboembolic complications should be included within cardiovascular sonography training. Ideally, fellows gain additional experience in echocardiography for patients who underwent heart surgery, to increase their confidence in assessing surgically implanted valve and aortic prosthesis, left ventricular assist devices, and transplanted patients. Training in echocardiography today should be conceived within the broader field of cardiovascular imaging. Depending on the structure of imaging branches within the teaching institution, fellows should work with a cardiovascular imaging specialist for 8 weeks in full-time. Analysing computed tomography scans, x-ray images, and pulmonary ultrasound in conjunction with a concise thoracic imaging course and participation in clinical imaging conferences will enable fellows to understand basic algorithms of image reporting, scan for overt findings, and integrate these with information obtained by other imaging modalities.

Complementary time dedicated to electrophysiology and devices is highly recommended before the cardiac ICU rotation, as well. After an introduction into diagnostics and programming of pacemaker and implantable defibrillator systems by a senior fellow or specialized consultant, fellows should work on regular elective cases for at least 8 weeks. Ideally, their knowledge should be expanded by shadowing (internal and external) cardioversions, device implantations, and participating in consultations on the cardiac ICU, thereby progressing towards a deeper insight into emergency management of arrhythmias and prevention in high-risk settings. Furthermore, completion of an advanced life support (ALS) course (e.g., ALS course certified by the European Resuscitation Council or the American Heart Association) should be mandatory. Ideally, larger cardiac ICUs should qualify their own physician and non-physician advanced cardiovascular life support (ACLS) instructors and conduct multidisciplinary training courses for ACLS providers. For example, a 6-member course can consist of one interventional cardiologist, two cardiology fellows/trainees from cardiac ICU, two ICU nurses and one catheterization laboratory nurse. Such training can be extremely fruitful and important for team building in general and understanding of the roles taken by different team members/professions. Fellows should use this opportunity to refine their knowledge of emergency care algorithms and practice their leadership role in a protected environment with a benign feedback culture. This may already be expanded on by early participation in morbidity and mortality conferences, where potential pitfalls are discussed openly, and preventive measures are established.

## An updated core curriculum for training in critical care cardiology

The 2015 COCATS 4 statement on Critical Care Cardiology Training (8) listed basic competences that should be acquired during a 3-year cardiovascular fellowship program (*Level I* training). In theory, fellows with a specific interest in CCC could satisfy optional training elements by extending their ICU exposure by 3–6 months during the standard fellowship period (*Level II* training). However, the definition of these elements remains vague owing to the limited experience with the suggested educational pathway. Advanced training modules for CCC sub-specialization are conceptualized within an additional 1-year fellowship program (*Level III* training). On behalf of the European Society of Cardiology (ESC), the 2014 Association for Acute CardioVascular Care (ACVC) Core Curriculum (9) also defined main objectives, knowledge, skills, and behaviours/attitudes across 20 key categories pertaining to acute cardiovascular diseases and other central didactic topics for CCC sub-specialization. To meet these training requirements, the ACVC stipulates at least month-long rotations in anaesthesia, respiratory medicine, and nephrology, in addition to a total of 18 months in a specialized cardiac ICU (6 months during cardiovascular fellowship and 12 months thereafter), as well as 3 months in a general medical ICU. While the curricula proposed by the ACVC, the international Competency Based Training programme in Intensive Care Medicine for Europe (CoBaTrICE) (10), and other committees, provide detailed prerequisites for independently managing (cardiac) ICU patients, there are no widely accepted, comprehensive standard curricula specifying how CCC training elements can be tailored to the various stages of a fellow's career.

In this chapter, we propose three sets of core training components (Tables 1–3), that have been compiled based on the abovementioned guideline recommendations, local training curricula at the authors respective institutions, and the collective opinion of CCC program directors, consultant critical care cardiologists, and cardiovascular fellows currently pursuing CCC training. While there is undoubtedly some overlap between the suggested categories, Tables 1–3 attempt to distinguish core elements of patient care, medical knowledge, practical skillset, competences in systems of care, and professionalism within basic level CCC training (Table 1), contrasting them with core competences in general intensive care medicine (Table 2), and advanced-level CCC training (Table 3).

**Table 1:** Basic level core competences in critical care cardiology.

Medical knowledge and skillset
Pathophysiology, diagnostic criteria, and basic treatment options of cardiogenic shock and associated syndromes depending on different underlying aetiologies: - Acute myocardial infarction and potential mechanical complications - Acute decompensated heart failure - Fulminant myocarditis - Acute decompensated valvular diseases - Pericardial tamponade - High-risk pulmonary embolism - Pulmonary hypertension - Aortic dissection - Acute arrhythmias
Pathophysiology, diagnostic criteria, basic treatment options, and outcomes in patients with fulminant infective endocarditis
Clinical pharmacology focussing on indications, contraindications, pharmacodynamics, and associated risks of: - Antiarrhythmic medications - Vasoactive/inotropic medications, calcium-sensitizers - Anticoagulant/antiplatelet/fibrinolytic medications - Antihypertensive medications including agents used for pulmonary hypertension - Heart failure medication - Diuretics - Sedatives, analgesics, and neuromuscular blocking agents - Antimicrobial agents
Basic understanding of indications, contraindications, and associated risks of interventional and surgical cardiac procedures: - Electrophysiological procedures for supraventricular/ventricular tachycardias - Percutaneous coronary interventions, chronic total occlusion PCI, bypass surgery - Surgical treatment of mechanical complications - Ventricular assist device implantation - Transcatheter aortic valve replacement, surgical valve replacement/reconstruction - Aortic surgery - Transcatheter tricuspid/mitral valve reconstruction/replacement, surgical replacement/reconstruction of atrio-ventricular valves - Transcatheter left atrial appendage occluder implantation - Transcatheter closure device implantation for inter-ventricular/inter-atrial shunts
Indication for heart transplantation, basic understanding of immunosuppressive treatment
Assessment and general management of post-operative patients
Pathophysiology, diagnostic criteria, differential diagnoses, basic treatment options, and outcomes of acute neurologic disorders
Pathophysiology, diagnostic criteria, differential diagnoses, basic treatment options, and outcomes of acute renal, acid-base, and electrolyte disorders
Pathophysiology, diagnostic criteria, differential diagnoses, basic treatment options, and outcomes of acute gastrointestinal disorders
Detection and management of peripheral and abdominal compartment syndromes
Patient care
Structured assessment of medical history and physical examination in an intensive care setting
Basic assessment of hemodynamic instability and respiratory dysfunction
Performing advanced cardiac life support according to standard algorithms
Basic understanding of post-resuscitation care
Understanding of emergency antiarrhythmic treatment options
Indication and interpretation of non-invasive and invasive hemodynamic monitoring
Indication and interpretation of laboratory testing including blood gas analysis
Management of hypertensive emergency/urgency
Indication and interpretation of radiographic imaging in liaison with imaging specialists
Indication for and basic interpretation of diagnostic left/right heart catheterization
Sedation management, analgesia, and neuromonitoring
Comprehensive fluid management
Management of bleeding or thromboembolic events
Management of hyperthermia, infection, and sepsis in liaison with microbiology specialists
Management of acid-base disorders
Comprehensive management of nutrition
Basic understanding of indication, contraindication, and associated risks of pharmacological and mechanical circulatory support options
Indication, contraindication, and associated risks of non-invasive and invasive respiratory support, basic weaning algorithms, indication of tracheostomy
Diagnostic criteria and management of acute kidney injury, indications, contraindications, and associated risks of renal replacement therapy
Prophylaxis and basic management of bleeding and thromboembolic complications
Management of multimorbid and elderly patients
Understanding principles of palliative care
Procedural skills
Insertion of arterial and central venous lines including large-bore catheters for renal replacement therapy
Performing cardioversion, defibrillation, and ventricular overdrive pacing
Transvenous pacemaker insertion, performing transthoracic cardiac pacing
Performing point-of-care echocardiography
Echocardiographic assessment of ventricular function, valvular function, and pericardial effusion
Basic sonographic assessment of vascular access sites, lungs, pleura, abdominal organs, and free abdominal fluid
Safe administration of blood products according to standard algorithms
Performing endotracheal intubation
Performing thoracocentesis and paracentesis
Placement of nasogastric tube
Obtaining appropriate microbiological samples
Performing basic vascular ultrasound
Transportation of stable patients outside the cardiac ICU
System of care competences
Understanding ICU admission criteria
Working in a multidisciplinary environment
Participation in M&M conferences
Participation in quality of care and safety training
Participation in institutional infection control and isolation training
Professionalism
Respectful interaction with all ICU team members
Demonstrating commitment to high-quality care
Self-reflection regarding the limitations of own knowledge and skill
Prioritizing and organizing clinical responsibilities
Performing adequate documentation
Communicating goals of care to patient and family of different backgrounds
Sensitivity to patient's wishes and spiritual/cultural background, particularly in end-of-life decisions
Obtaining informed consent
Respecting the patient's privacy and autonomy
Addressing psychosocial issues of patients and families
Understanding ethical/legal implications of withdrawal of care
Engaging in continuous self-directed learning through seminars, web-based/simulation-based training programs, and teaching sessions embedded in scientific conferences

ICU, intensive care unit; M&M, morbidity and mortality; PCI, percutaneous coronary intervention.

**Table 2:** Core competences in general intensive care medicine.

Medical knowledge and skillset
Advanced assessment and management of post-operative patients
Pathophysiology, diagnostic criteria, hemodynamic implications, differential diagnoses, treatment options, and outcomes of anaphylactic, septic, hypovolemic, haemorrhagic, and obstructive shock
Pathophysiology, diagnostic criteria, treatment options, and outcomes of multi-organ dysfunction
Pathophysiology, diagnostic criteria, differential diagnoses, treatment options, and outcomes of acute respiratory disorders; indications, contraindications, and associated risks of VV-ECMO therapy
Pathophysiology, diagnostic criteria, differential diagnoses, treatment options, and outcomes of acute endocrine disorders
Pathophysiology, diagnostic criteria, differential diagnoses, treatment options, and outcomes of acute intoxication
Pathophysiology, diagnostic criteria, differential diagnoses, treatment options, and outcomes of acute peripartum complications
Assessment and general management of trauma patients
Patient care
Advanced management of resuscitation
Advanced circulatory management, optimization of preload and afterload using different medical options, balanced fluid management, diuretics, and renal replacement therapy
Interpretation of complex electrocardiography findings
Advanced management of respiratory support including different ventilation modalities and (prolonged) weaning algorithms
Advanced anti-microbial management, antibiotic stewardship, particularly in patients with intrinsic or acquired immunodeficiencies
Integrating multiple imaging modalities into clinical context
Management of high-volume fluid and blood transfusions and associated risks
Interpretation of brain death diagnostics, assessment of organ donation criteria
Participation in multidisciplinary withdrawal-of-care decisions
Advanced management and prevention of pain and psychological distress
Procedural skills
Performing advanced thoracic ultrasound
Performing advanced abdominal ultrasound
Performing lumbar puncture
Performing tracheotomy
Performing bedside fiberoptic laryngotracheobronchoscopy
System of care competences
Engaging in clinical consultation
Coordinating referrals and patient triage
Management of retrieval and transfer of ICU patients from other units/hospitals
Understanding ICU discharge criteria
Engaging in teaching activity for junior fellows
Critical application of (inter)national and local guidelines and protocols
Professionalism
Assuming a leadership role and striving for holistic patient care
Promoting effective multidisciplinary team-working
Communication of complex case summaries to team members and other health care professionals
Implementation of new guidelines and scientific papers into clinical practice
Identifying potential risks for ICU staff and promoting adequate safety measures
Giving structured feedback to ICU team members
Establishing a constructive culture of dealing with medical errors
Evaluating cost-effectiveness of advanced treatment options
Participation in international conferences

ICU, intensive care unit; VV-ECMO, veno-venous extracorporeal membrane oxygenation.

**Table 3:** Advanced level core competences in critical care cardiology.

Medical knowledge and skillset
In-depth knowledge on pathophysiology, diagnostic criteria, differential diagnoses, advanced treatment options, and outcomes of structural heart diseases and cardiomyopathies
In-depth knowledge on pathophysiology, diagnostic criteria, differential diagnoses, advanced treatment options, and outcomes of primary arrhythmic disorders
In-depth knowledge on pathophysiology, diagnostic criteria, differential diagnoses, advanced treatment options, and outcomes of left-ventricular, right-ventricular, and bi-ventricular cardiogenic shock, associated syndromes, and underlying aetiologies
Pathophysiology, diagnostic criteria, treatment options, and outcomes of cardiogenic shock and other critical care scenarios in patients with inherited heart diseases (i.e. hypertrophic cardiomyopathy, arrhythmogenic right ventricular cardiomyopathy, Long-QT-syndrome)
Pathophysiology, diagnostic criteria, treatment options, and outcomes of cardiogenic shock and other critical care scenarios in special patient populations (i.e. HIV-positive patients, pregnant women, patients after heart transplantation, patients with left ventricular assist device, pulmonary hypertension, Takotsubo-cardiomyopathy, oncologic patients)
In-depth knowledge of indications, contraindications, and associated risks of the full spectrum of interventional cardiac treatment options in acute high-risk settings
Indications, contraindications, and associated risks of mechanical circulatory support for patients in advanced/refractory cardiogenic shock or cardiac arrest, i.e.: - VA-ECMO - IABP - Tandem-heart - Impella
Indications, hemodynamic implications, contraindications, and associated risks of different LV-venting modalities during VA-ECMO therapy
Assessment and management of patients admitted to the cardiac ICU after extracorporeal cardiopulmonary resuscitation
Patient care
Assuming leadership role during cardiopulmonary resuscitation and integrating emergency diagnostics to formulate management plan
Management of patients receiving complex interventional procedures including coronary interventions, valvular interventions, and device implantation
Detection and management of multi-organ failure
Indication and interpretation of advanced hemodynamic monitoring (i.e. PICCO, thermodilution)
Indication for and comprehensive interpretation of diagnostic left- and right heart catheterization as well as various radiographic imaging modalities
Advanced management of patients receiving MCS devices including VA-ECMO, Tandem-Heart, IABP, Impella
Management of patients in advanced heart failure stages including eligibility criteria for left ventricular assist device implantation and high-urgency listing for heart transplantation
Emergency assessment, post-operative care, and complication management of patients with durable left ventricular devices
Advanced post-resuscitation care including targeted temperature management
Procedural skills
Performing advanced point-of-care echocardiography
Performing bedside transoesophageal echocardiography
Performing advanced vascular ultrasound
Performing pericardiocentesis
Comprehensive assessment of arrhythmias recorded by implantable cardiac devices, function assessment and adjustment of the settings of bradycardia pacemakers as well as anti-tachycardic function of implantable cardioverter defibrillators
Cannulation, setup, maintenance, and decannulation of VA-ECMO and Tandem-Heart devices
Insertion, maintenance, and explantation of IABP and Impella devices (excluding surgical implantation and explantation of Impella 5.0 and 5.5)
System of care competences
Coordinating and leading multidisciplinary daily rounds
Coordinating and supervising intra- and interhospital transfers of cardiac ICU patients
Initiating and moderating M&M conferences
Assessment of mortality and other relevant outcome variables using standard risk prediction models and (inter)national registries
Professionalism
Gradually assuming responsibility for the full spectrum of CCC patients
Promoting efficiency and effectiveness of patient care within the CCC team
Shared decision making with patients, families, and other health care professionals
Engaging in teaching and continued scholarly activity

IABP, intra-aortic balloon pump; ICU, intensive care unit; M&M, morbidity and mortality; PCI, percutaneous coronary intervention; PICCO, pulse index continuous cardiac output; VA-ECMO, veno-arterial extracorporeal membrane oxygenation.

Table 1 outlines competences that are needed for the trainee's first integration into a cardiac ICU team, representing the initial point of contact with critically ill cardiac patients. Irrespective of their chosen sub-specialty, these core CCC training elements are relevant to all cardiovascular fellows for gaining a full understanding of cardiac emergency care. Table 2 depicts a broader set of core competences in intensive care medicine, focussing on the management of a greater spectrum of acute medical illnesses, trauma, and post-operative care as well as a broader practical skillset and leadership qualities essential for assuming responsibility for patients in a general (surgical or medical) intensive care setting. Lastly, specific training elements for specialization in CCC are presented in Table 3. This set of core training components is incomplete by nature, and the allocation of individual elements warrants further discussion. Nevertheless, the suggested framework may function as a handrail for the development of CCC sub-specialization programs and for cardiovascular and intensive care medicine fellows aspiring to advance in this direction.

Structured assessment of the trainee's clinical performance is essential to boost compliance with educational benchmarks and ensure the highest standards of training and professional development. Personal evaluation should also consider scientific achievements and continued assessment of quality of care after specialization. Beyond that, the need for quality assessment extends to the specialization program itself. Intensive care societies and program supervisors should strive for establishing key performance indicators, such as the number of training positions per year, the number of graduates per year, dropout rate, and measures of flexibility, as well as indicators of institutional conditions, that help identify the need for structural optimization, and improve comparability and measurability of training outcomes on a national/international level.

## Sub-specialization pathways and institutional preconditions

**Figure 3:**
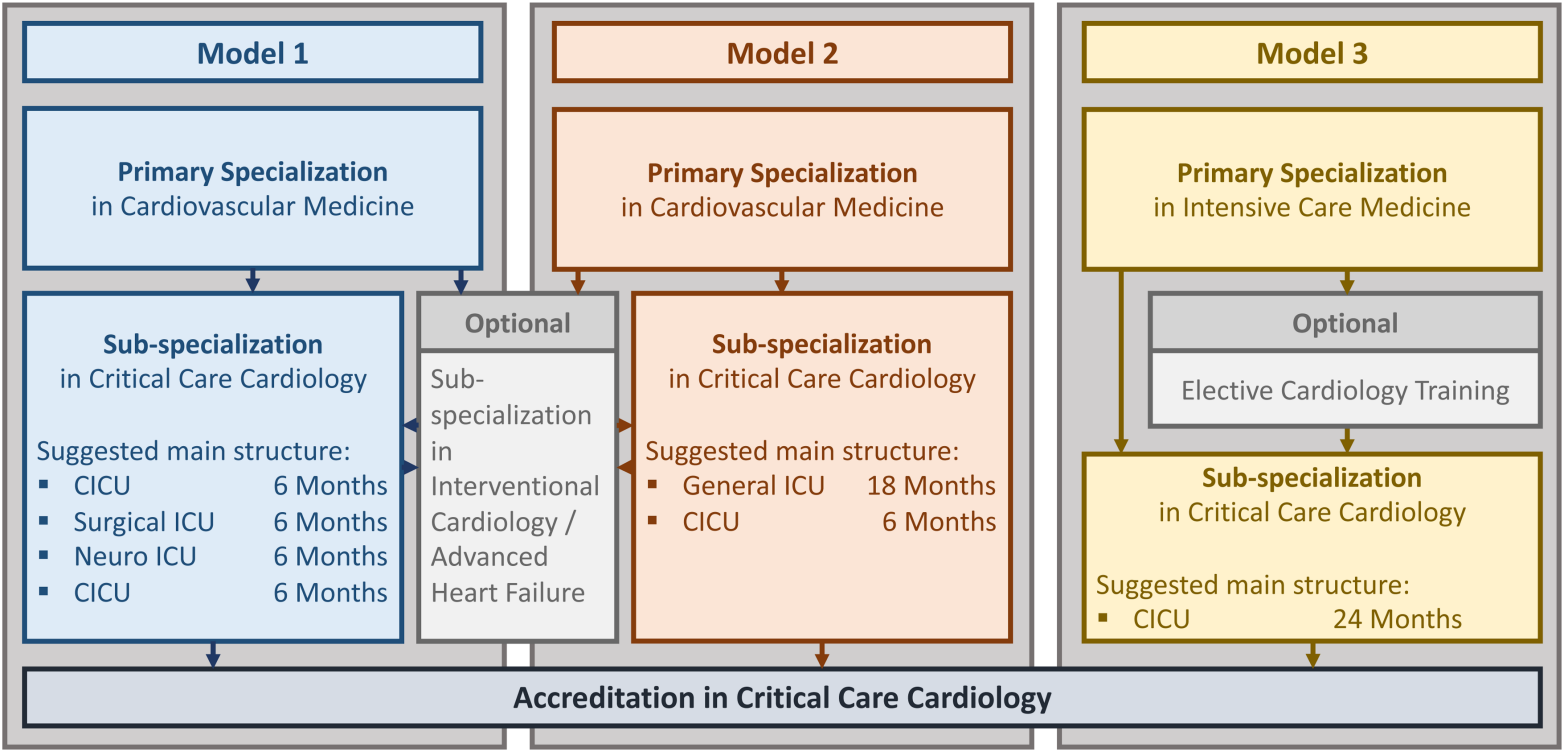
Sub-specialization tracks in critical care cardiology. CICU, cardiac intensive care unit; ICU, intensive care unit.

The challenge of defining training targets is not the only issue at hand. Educational guideline committees and program supervisors must also navigate the delicate balance between incorporating the evolving skillset and knowledge in CCC and minimizing specialization durations. Inspired by training pathways at the Ludwig-Maximilians-University (LMU) hospital and other international accreditation tracks, we propose three adaptable models for sub-specialization in CCC (Figure 3) in the following section (6, 11–13).

Models 1 and 2 are based on completion of a fellowship in general cardiovascular medicine including at least 6 months of ICU training, which reflects the application requirements for CCC sub-specialization set forth by the ACVC. In conjunction with the knowledge acquired during rotations in cardiac imaging, electrophysiology, and to the catheterization laboratory, the content of Table 1 represents key milestones for the cardiovascular fellow's integration into the cardiac ICU roster as a junior team member. For example, trainees must gain a common understanding of cardiac interventions that enables them to systematically screen for and manage complications on a basic level (5, 14). Also, the hemodynamic principles of cardiogenic shock along with treatment concepts and underlying diseases, as well as general handling of mechanical circulatory support (MCS) devices are nowadays core elements of training in cardiovascular medicine (14). By contrast, the in-depth knowledge on indications and risks of interventional cardiac procedures, such as emergent coronary interventions, transcatheter aortic valve replacement (TAVR), interventional mitral valve repair, mechanical thrombectomy, implantation of transcatheter closure devices, and ablation of ventricular tachycardias, that are required for decision-making in high-risk patients with hemodynamic instability go beyond what can be expected from early career fellows (15–20). Similarly, comprehensive management of cardiogenic shock patients including advanced understanding of hemodynamics and device-based treatment options requires a profound familiarity with current evidence on MCS and an advanced skillset for emergent troubleshooting in case of deterioration or device-associated complications (21–23). Models 1 and 2 envision the highest level of qualification (Table 3) to be acquired during an additional 12-month (Model 1) or 6-month (Model 2) cardiac ICU rotation as part of the CCC sub-specialization program. The concept of Model 1 further allows for a 6-month rotation in a surgical and neurologic ICU, respectively, to extend the trainee's general intensive care knowledge and skillset (Table 2). By contrast, formal training time in a general ICU according to Model 2 would be an 18-month period followed by 6 months of time dedicated to the cardiac ICU. When the 24-month sub-specialization program is completed successfully, trainees should have gained the knowledge and experience needed to act independently as a consultant cardiac intensivist and assume a leadership role within the multidisciplinary cardiac ICU team. In theory, programs adopting Models 1 or 2 could integrate advanced CCC training content (Table 3) with other advanced fellowship programs, e.g., interventional cardiology or heart failure (24). Compared to CCC, heart failure sub-specialization training typically covers additional aspects of long-term patient management, such as evaluation for heart transplantation or ventricular assist devices, and long-term follow-up. Combining training in CCC and heart failure could leverage the significant overlap in theoretical knowledge and practical skillset between these sub-specialities while strengthening the candidate's abilities to develop individual treatment concepts for the acute, intermediate, and chronic phase.

The structure of Model 3 differs from Models 1 and 2 regarding the basic clinical training that precedes sub-specialization in CCC. This pathway features a fellowship in general intensive care medicine, which is often designed in conjunction with training in anaesthesiology and pulmonary medicine, followed by 24 months of time dedicated to sub-specialization in CCC. For example, the current stage 1 postgraduate program in intensive care medicine in the United Kingdom spans a total of 4 years subdivided into training blocks in anaesthesiology, internal medicine, and emergency care (25). While this type of initial clinical exposure strengthens core qualifications in intensive care such as endotracheal intubation and ventilation management (Table 2, elements of Table 1), integrating the content of general cardiology training, which is indispensable for managing cardiac ICU patients, is more challenging. Within their internal medical rotations, trainees should focus on acquiring basic knowledge on the management of patients with structural heart disease and arrhythmias. Working in emergency care and in cardiac anaesthesiology provides learning opportunities about acute cardiovascular illnesses and treatment algorithms and could further improve the fellow's ability to assess arrhythmias and perform transthoracic and transoesophageal echocardiography. Program directors should encourage fellows interested in CCC to seek mentorship from within the cardiovascular department and engage in extra-curricular interdisciplinary seminars. Nonetheless, sub-specialization programs designed for candidates holding board certification in intensive care medicine must be adaptable to accommodate the trainee's individual level of experience in the field of cardiovascular medicine. Model 3 suggests a minimum of 24 months spent in the cardiac ICU, which may be complemented by rotations to the catheterization laboratory or heart failure unit. This approach could allow for a personalized roadmap leading to the acquisition of core competences in basic and advanced CCC (Table 3, elements of Table 1).

All three models provide a framework for integrating the extensive training content into personalized sub-specialization tracks, which potentially allow for interruption of training, rotations/transitions to non-tertiary institutions, and hybridization with other sub-specialization programs. Generally, many crucial aspects of training are contingent on team composition and organization of the CCC program established by the teaching institution. To ensure patient safety and efficacy of training, CCC sub-specialization programs should be situated in accredited tertiary hospitals and overseen by an experienced cardiac intensivist with the requisite national teaching qualification. Integrating specialists in interventional cardiology, cardiac surgery, electrophysiology, cardiac imaging, microbiology, and pharmacology into recurrent meetings and cardiac ICU rounds presents trainees with opportunities to gain a more comprehensive understanding of multidisciplinary treatment concepts and liaise with experts to enhance their skillset. By promoting scientific endeavours, institutions will contribute to academic progress and foster evidence-based medicine as well as life-long learning.

## Conclusion

To keep pace with the rapidly changing field of CCC, intensive care societies should establish an updated training concept, that encompasses core training elements spanning all career stages, different educational pathways, and formal institutional standards. Harmonizing regional training models into a comprehensive educational framework can provide valuable guidance for program directors and help fulfilling the needs of future critical care cardiologists. Ultimately, the versatility and holistic nature of training will play a pivotal role in shaping the success of the next generation of experts in the field as they work toward enhancing patient safety and achieving favourable outcomes.

## Data Availability

The original contributions presented in the study are included in the article/Supplementary Material, further inquiries can be directed to the corresponding author.
